# “Mom, what are you painting?” Virtual reality assessment of mother–daughter dynamics through joint art-making

**DOI:** 10.3389/fpsyg.2025.1597831

**Published:** 2025-10-15

**Authors:** Aya Bernhard, Liat Shamri Zeevi

**Affiliations:** Ono Academic College, Kiryat Ono, Israel

**Keywords:** virtual reality, JPP, mother-daughter relations, dyadic therapy, art-based intervention, art therapy

## Abstract

**Introduction:**

This study explored the use of the Virtual Reality Joint Painting Procedure (VR-JPP) to examine the relationship dynamics between mother-daughter dyads in middle childhood (ages 9 to 12). The research focused on art-based interventions conducted in the virtual reality space.

**Methods:**

Interviews were conducted with 16 mothers who participated in the VR-JPP.

**Results:**

The interviews yielded three main themes: dyadic communication in the virtual reality space, the advantages and disadvantages of three-dimensional painting in virtual reality, and the tripartite relationship between the therapist, mother, and daughter in the therapeutic space. The findings suggest that virtual reality fostered an innovative therapeutic space that encourages freer and more open interactions between mothers and daughters using avatars that enable unmediated emotional expression. The virtual space allowed the dyads to focus on emotional and creative communication and increased their grasp of relationship dynamics.

**Discussion:**

The virtual space allowed the dyads to focus on emotional and creative communication and increased their grasp of relationship dynamics. These findings contribute to the literature on the uses of art in the virtual reality space from the perspective of mothers.

## Introduction

This study explored mother-daughter relations in middle childhood over the course of an art-based intervention implementing a novel adaptation of the Joint Painting Procedure (JPP) for virtual reality (VR).

In girls, middle childhood occurs between the ages of 9 to 12 and corresponds to a crucial pre-adolescent period of psychosocial development (Gilmore and Meersand, 2023). During middle childhood, girls experience a significant increase in their cognitive abilities and improvement in their social skills, as well as a burgeoning sense of independence. Mother-daughter relations undergo notable changes, where girls seek more autonomy while relying on maternal support for emotional security. Mothers often play a major role in shaping their daughters’ self-esteem, body image, and future relationships. The quality of the mother-daughter relationship can thus significantly affect the daughter’s psychological well-being and her social adjustment in the present and future (Rodgers et al., 2020a). As girls approach adolescence, mother-daughter dynamics can become more complex, with a potential escalation in conflicts and emotional intensity (Lee and Mun, 2022). Maintaining open communication and emotional availability during this period can foster a strong foundation for navigating the challenges of adolescence (Friedman M., 2013). Studies (Fisher Grafy, 2015; Brumariu and Kerns, 2022) have shown that parental support and active parent–child interactions during middle childhood can positively influence the later stages of identity development, in particular the processes of exploration and commitment. These developmental challenges highlight the importance of supportive interventions aimed at strengthening parent–child relationships during this stage, including art-based approaches.

### Art-based parent–child therapy

Theories of dyadic attachment are based on the premise that the parents’ personality structure as well as the parent–child relationship can affect the child’s behavioral traits and emotional needs (Bornstein et al., 2022). Kaplan (2019) stressed that the therapist can play a key role in assessing relationships through parent–child interactions in a clinical setting. In this context, the therapist is considered the analytic third, who experiences their interactions as they unfold explicitly and implicitly in the ‘here and now’. These interactions within the parent, child, and therapist triangle contain reconstructed contents from previous relationships that mutually influence one another.

Art-based dyadic methods have increasingly been used to enhance relational dynamics since they can provide a shared creative medium for both parents and children. These art-based approaches presents opportunities for both verbal and non-verbal communication, encourages symbolic play and projection, and creates a joint space where attachment patterns can be observed, reenacted, and potentially reshaped (Gavron and Mayseless, 2015; Shamri-Zeevi and Kedem-Sarrabia, 2023). Art therapy combines artmaking with emotional expression and psychotherapy. It enables clients to explore their mental processes and may contribute to resolving personal and interpersonal conflicts that can lead to greater wellbeing (Malchiodi, 2012; Rubin, 2010). The art therapist suggests specific art-based interventions with dedicated materials and techniques that allow for conscious and unconscious expression in the dyadic relationship (Shamri-Zeevi and Regev, 2021). Gavron (2017) emphasized the importance of identifying the child’s emotional needs in the dyadic relationship through observations of the creative processes and the dyadic product that are considered symbolic expressions that reflect the pillars of the child–parent relationship (Gavron, 2013, 2017; Shamri-Zeevi et al., 2015).

Parent–child nonverbal interactions during painting can also reveal the implicit aspects of the relationship. This diagnostic information is embodied in the artmaking itself and in the symbolic visual contents that arise from it. Joint artmaking both reflects and affects the relationship dynamics, while at the same time providing a safe space for self-expression where the participants can reflect on their relationships creatively and in real time (Gavron et al., 2022). Gavron’s Joint Painting Procedure (JPP) is an arts-based guide to the assessment of nonverbal expressions of the parent–child relationship during joint painting activities (Gavron and Mayseless, 2015). The JPP is designed to document each participant’s individual inner representations, as well as the relationship between parent and child as manifested in the artmaking process and product.

### Artmaking in virtual reality

Recent technological developments now allow these art-based methods to be extended to immersive virtual environments, thus opening new avenues for the for assessment and therapy of parent–child relationships. VR constitutes a technological and interactive three-dimensional (3D) environment where multiple participants can share a virtual space generated by stereoscopic 3D displays that create a realistic 360-degree sense of depth and immersion (Alsem et al., 2023). This technology makes it possible to experience a different reality by creating an illusion where what the person sees, and sometimes also hears and feels, is replaced by a visual simulation of a realistic or imaginary digital environment (Piryankova, 2015).

VR in art therapy thus constitutes a cutting-edge approach to mental health therapy that offers unique opportunities for technology-based creative expression and therapeutic interventions (Hadjipanayi et al., 2023; Perez, 2024). VR-based artmaking encompasses a wide range of creative possibilities that include 3D techniques, as well as realistic or imaginary elements from the world of art, thus combining elements such as line, form, patch, and color from painting and sculpture “classically” used in art therapy sessions with digital elements (Hadjipanayi et al., 2023; Kaimal et al., 2020; Shamri-Zeevi, 2021). Artmaking in the digital medium involves virtual rather than physical material. The painting is created in 3D, so that it can be viewed from multiple angles, including from within the work. The virtual space allows “unrealistic” features, since the artwork is not subject to the laws of physics, and the material can include elements that move in infinite space. When difficult emotional materials emerge, the client can control the situation by removing the VR headset (Shamri-Zeevi, 2021).

Previous studies have shown that VR can foster creativity in three-dimensional painting environments and expand opportunities for symbolic expression (Haeyen et al., 2021b; Kaimal et al., 2020). In clinical contexts, VR-based art therapy has been associated with reduced anxiety, improved emotional processing, and enhanced engagement in treatment (Hacmun et al., 2018; Shamri-Zeevi, 2021). More recent work has highlighted the potential of multi-user VR environments in strengthening the therapeutic presence and relational engagement (Jans et al., 2025; Li and Shen, 2022). Systematic reviews have emphasized the importance of developing accessible and low-cost VR applications for wider therapeutic use (Best et al., 2022).

To date however, few studies have explored the use of VR in mother-daughter art therapy. The present study addressed this gap by adapting the Joint Painting Procedure (JPP) to a virtual reality format, thereby contributing to the emerging literature on dyadic art-based interventions.

## Materials and method

This study explored mother-daughter relations in middle childhood while mother-daughter dyads were engaged in a VR version of the Joint Painting Procedure. This study employed an interpretivist approach with symbolic interpretation which recognizes that the artmaking process and participants’ experiences contain multiple layers of meaning that require an interpretive rather than a purely descriptive analysis (Liu and Dance, 1995). This framework acknowledges that meaning emerges through the interaction between researchers, participants, and the symbolic content of their artistic expressions (Rasmussen, 2012).

### Sample

This study was based on interviews conducted with 16 mothers of daughters aged 9 to 12. They were recruited through social media, parent groups, and personal networking. This exploratory study examined nonclinical participants functioning within the normal range. The only inclusion criterion was the daughters’ age (9–12 years) (see Table 1). The participants were all from central Israel, and in their 40s (*M* = 40), with a bachelor’s degree as their mean level of education. The mean age of the daughters was 10 (*M* = 10.28). On average, the mothers had little or no prior experience with VR, and their daughters had engaged in VR sessions 2–3 times gaming previously.

**Table 1:** Demographics of the mother-daughter dyads.

Dyad	Mother’s age	Schooling	Previous VR experience	Daughter’s age	Previous VR experience	Children in the family	Residential area
1	40+	MA	3	9.5	5	3	Center
2	40+	BA	0	9.5	1	3	Center
3	30–40	BA	0	11.5	0	3	Center
4	40+	Vocational diploma	1	9.5	2	3	Center
5	40+	BA	1	10	3	3	Center
6	40+	BA	1	12	5	3	Center
7	40+	MA	1	11	1	3	Center
8	40+	MA	0	9.5	2	2	Center
9	30–40	MA	0	11	0	3	Center
10	30–40	MA	0	11	2	2	Center
11	40+	MA	0	10	1	3	Center
12	40+	MA	0	11	1	3	Center
13	40+	BA	3	10	3	2	Center
14	40+	Vocational diploma	1	10	1	2	Center
15	40+	BA	2	9	2	3	Center
16	40+	BA	1	10	5	3	Center

### Tools

This qualitative study was based on semi-structured interviews with mothers after they took part in the VR JPP. The questions were formulated in advance but were written to be open-ended enough to permit the respondents to expand on content in response to queries from the researcher.

#### Joint painting procedure

The JPP is a validated assessment tool implemented in clinical interventions analyzing dyadic relationships (Gavron, 2013, 2019). The standard JPP assessment has five stages that consist of making a two-dimensional (2D) painting on a shared sheet of poster-sized paper. In the first stage, the parent and child paint separately side by side, after defining their personal areas with a pencil line. The second stage involves painting in the personal area on a chosen topic with gouache/tempera paints. In the third stage, a frame is drawn around the personal painting with its own shape and texture. In the fourth stage, parent and child are asked to paint a path from their personal frame to the frame of their partner; the path is made by each and can be produced in any medium. In the fifth stage, the parent and child paint together in the shared space, i.e., the area outside the designated personal areas and connecting paths. Once completed, the painting is observed, the experience discussed, and a joint title and story are formulated. In the current study, the mother-daughter dyads engaged in a joint virtual space that included the researcher while following the five stages of the JPP procedure.

#### Materials

The VR equipment was composed of three Meta Quest 2 wireless VR headsets, one for each participant, and two two-touch controllers for each participant (one for each hand). MultiBrush, a third-party multiplayer VR painting application based on Google’s open-source Tilt Brush code was used. This application allows several participants to paint simultaneously in virtual 3D space. Users manipulate self-avatars that move freely in a predetermined secure area and can interact with others in the same environment. As a graphic representation tool, these avatars increase the users’ sense of presence and self-identification in the virtual environment. They can be personally adapted to take the form of a human, animal, or imaginary creature, and can produce hand, head, and body movements, facial expressions, and emotional reactions. The avatars emulate the movements and the participants’ expressions to increase communication effectiveness, enhance immersivity, and reduce cognitive overload (Hart et al., 2021; Rahman and Rahman, 2021; Freeman et al., 2020; Day et al., 2019; Lugrin et al., 2018).

A reflective journal was kept by the first researcher. Reflective journaling is a prime tool in arts-based research, particularly within the field of art therapy that allows the researcher-practitioner to examine subjective experiences and meaning-making processes throughout a study. By writing reflections alongside the artistic process, researchers can gain deeper insights into their own responses, biases, and emotional engagement, while also enhancing the interpretative validity of the research (Leavy, 2020; McNiff, 2008). This practice not only supports critical reflexivity but also bridges personal creativity with scholarly inquiry, thus enriching both clinical and academic perspectives (Knowles and Cole, 2007).

#### Instructions

Prior to the VR experience, each mother-daughter dyad was taught how to use the headset and the right and left controllers on the MultiBrush application. The left controller is for color palettes, brushes, textures, and effects. The right controller has the eraser tool and is also used to move in virtual space via teleport. After a brief familiarization session, the dyads engaged in the structured joint painting procedure (JPP) which was modified slightly to be appropriate for VR. At the end of the artmaking, a semi-structured interview was conducted with the mothers. The daughters, who remained in the room, occasionally provided technical assistance to their mothers with the VR equipment.

#### Semi-structured interview

A semi-structured open-ended in-depth interview was written specifically for the purposes of this study. The interview comprised a series of predetermined questions that could be adapted as a function of the dynamics with the interviewee; specifically, the interviewer could change the order of the questions and add further questions as the interview unfolded (Routledge Creswell, 2014; Patton, 1990). The interviews probed how the mothers perceived the meaning and impact of the JPP intervention, mother-daughter relations, perceived parental efficacy, the integration of VR, and the impact of the procedure on the mother-daughter relationship. The interviews with the mothers were conducted by the first author, and included questions such as: Did you discover anything new about your daughter that you did not know previously? Did you feel at some stage of the painting that you would like to intervene in your daughter’s painting or help her out?, What happened in your joint experience that would not have happened in everyday life?, Which was easier for you – the joint painting or the separate painting?

### Procedure

Mother-daughter dyads (a non-clinical sample of daughters aged 9 to 12) were recruited and provided their formal written consent to participate in the study. The dyadic encounter took place in a specially designated space. The participants completed a short demographic questionnaire, and the researcher provided an explanation on how the VR tool is used. The familiarization with the VR technique was followed by the structured VR-JPP procedure. This procedure was recorded in video, audio and still images through the VR headset (Bauer and Gaskell, 2000). After the experience, semi-structured interviews were held with the mothers. During the art-based experience in the virtual space, while wearing the VR headset, the participants were represented by motion avatars that used their voice and could be identified by their movements. The avatars were identical, with different names for the mother, daughter, and researcher. No eye contact could be established, and no facial expression could identify the avatars. The avatar’s height was adjusted to the participant, to easily differentiate between mother and daughter. Note as well that the therapeutic setting during the VR-JPP procedure provided a secure and controlled environment with double boundaries: the physical boundaries of the room, and the virtual boundaries defined by what is known as the Guardian system. The Guardian system, which is integrated into VR headsets such as Meta Quest, allows users to establish virtual boundaries in their physical space. These boundaries appear visually when users approach the edges of their predefined VR area and help prevent collisions with physical objects or walls (XR Today, 2023).

### Data processing

Data analysis was based on a narrative and a phenomenological approach (Kapitan, 2010; Spector-Mersel, 2010). This approach, known as inter-coder reliability, reduces individual bias and ensures the consistency of the findings (O’Connor and Joffe, 2020). Triangulation was achieved by integrating the three data sources (VR session recordings, still images of the artworks, and mothers’ interviews). In this study, the researcher’s dual role as both a participant within the VR environment and the primary data analyst was actively managed through strategies including ongoing reflexive journaling and external supervision. These methods were critical to ensuring that personal involvement did not compromise the objectivity or integrity of the findings. The interviews were analyzed following the principles of thematic analysis, to identify and define major themes, ideas, and interventions (Charmaz, 2006). The data analysis was based on the three stages recommended by Strauss and Corbin (1990). In the first stage of the analysis, the open coding stage, where the meaning categories are extracted from the data, the transcripts were read several times to enhance familiarity with the text, and the major themes were marked. In the second, axial coding stage, the categories were processed several times until they contained all the information arising from the interviews. In the final selective coding stage, where the “story” was extracted, all the interviews were read once again to reexamine the compatibility of the information units with the major themes and organize them into a structure where each category was linked to the others (Sabar-Ben Yehoshua, 2016). The analysis of the VR experience was made up of a comprehensive observation of explicit (verbal interaction) and implicit components (body gestures, use of textures, colors, common elements, and others) as described in the Findings section.

### Ethical approval

This study was carried out with sensitivity with respect to the interviewees and their relationship. The participants were guaranteed that their personal information would be kept fully confidential to protect their privacy. The participants signed an informed consent form and gave their consent to use the interviews, video recordings, audio recordings, and photographs for research purposes in all stages of the study. This study was approved by the Ethics Committee of the Academic College of Society and the Arts, No. ASA00645 (June 2023).

## Results

This section presents the three main themes derived from the interviews and the dyadic VR experiences when implementing the VR-adapted version of the JPP assessment tool in a multiplayer painting application (see Table 2).

**Table 2:** Themes and sub-themes.

Theme	Sub-theme
1. Dyadic Communication and conflict in the VR Space	Verbal Communication -orientation, task-related dialog, affectionate terms, conflictual expressions, reflective discourse
	Contact-based Communication – The Immersive Body versus “Virtual” Physical Contact – virtual hugs and kisses, altered bodily perspectives, dancing, teleportation (hide-and-seek), distancing behaviors
	Interpersonal-Implicit (Unconscious) Communication in the Virtual Space – presence vs. separation, rebellious expression, cracks and transparency, erasing as separation or closeness, scribbling on mother, bubbles and enclosure
	Body Postures when Painting in VR – closeness versus distance and emerging mother-daughter conflict– face-to-face vs. back-to-back, proximity and distance as symbolic positioning
2. 3D Painting in VR and Mother-Daughter Relations	The Three-Dimensional House – 2D vs. 3D houses, entering houses, shared houses, decorated houses, house as symbol of autonomy and relatedness
	Recurring Images and Integrating Text – flowers, suns, hearts, rainbows, use of names and titles, written expressions of belonging, pride, identity
	Mutual Emulation and Attachment Dynamics – daughters mirroring mothers, mothers adopting daughters’ motifs, symbolic negotiation of closeness and independence
3. The Immersive Therapist in the Dual Therapeutic Space	“Invisible” Presence in the Shared Space – avatar therapist often unnoticed, neutral presence not disturbing immersion
	The Immersive Therapist Role – analytic third within VR, observing transference and countertransference, facilitating projective processes
	Double Boundaries in the Therapeutic Environment – dual presence (in-person and avatar), neutrality enhanced by immersion, expanding implications for dyadic and group VR therapy

### First theme: dyadic communication and conflict in the VR space

Mother-daughter communication in the virtual space included conscious (explicit) verbal communication, as well as 3D auditory-movement artistic expression. The shared 3D VR space that included the researcher made it possible to observe the dynamics of the mothers’ and daughters’ movements toward and away each other in real time. The dyads spoke and used gestures continually throughout the VR experience, which enriched the interaction.

#### Verbal communication

Different types of verbal communication between mothers and daughters were observed. When the dyads began to explore the unfamiliar 3D space, verbal communication was used to convey information and find a shared location such as “Mom, where are you?” “Honey, press the teleport in my direction,” “Whoa, I’m behind you.” Verbal communication reappeared during the joint painting, mainly in questions related to choosing the topic: “What should we paint?” “What topic would you like?.” Throughout the virtual experience, dyadic communication was accompanied by considerable endearments by the mothers. In more conflictual segments, the mothers resumed their use of the daughter’s name. Unlike the mothers, the daughters mainly called the mothers “Mom”; a few used their mother’s given name.

In most of the dyads, the art-based virtual experience began with enthusiastic communication, with the verbal expressions conveying liberation such as: “Wowee,” “Mine came out cool.” A few dyads expressed disappointment and frustration: “Ohh Mom, we are getting too close to the paintings, I have to erase….”.

In later stages, once the techniques for working with the application were better acquired, verbal communication was characterized mainly by mumbling, with a decline in dyadic communication, as the dyads engaged in their individual artmaking. In Dyad 14, the mother sang out loud throughout the virtual experience. The app’s audio permits multiple speakers’ audios to transmit simultaneously; however, the daughter chose to remain silent when the mother sang, except when painting in the shared space. The mothers’ discourse during the VR differed from the post-experience interview. For example, in Dyad 5, the mother concentrated on the painting experience and declared throughout that it felt like being “high on drugs,” while her daughter constantly asked: “Mom, what are you doing? Do you know that you are visible?” (The mother moved in a strange way and did not respond to her daughter because she was immersed in her VR world). By contrast, in the interview the mother said:

…Each of us was involved with her own [work] and we were next to each other, we could engage in the same activity throughout, and both of us enjoyed., neither had to be there for the other… We were sort of equal. Not the role of mother and daughter. When she occasionally said: “Mom, look” I turned around… She was excited but she did not say that, so I could concentrate on myself.

During the artmaking, communication was experiential and emotional. The mothers and daughters expressed their feelings promptly and spontaneously. Statements such as: “I just want to stand and look and feel,” and “I’m going to get out in a big way, I’m anticipating a big disappointment,” reflected the emotions that emerged in real time in the VR space. During the interviews, the mothers analyzed their experience using a more cognitive and reflective language when discussing their role as a parent and their relationship with their daughter. Statements such as: “I understand that we do not spend enough time together” were suggestive of the complexity of the artistic experience. The VR elicited spontaneity and immediacy whereas the interviews facilitated more in-depth processing of the experience.

#### Contact-based communication – the immersive body versus “virtual” physical contact

**Figure 1:**
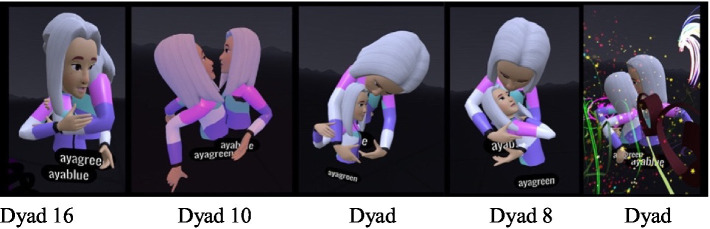
Virtual hugs and altered height.

While wearing the VR headset, the participants were represented by avatars that had their voices and were identified by their movements. The transition to the immersive world allowed for virtual “physical” contact that resembled actual contact but was not felt tangibly. This contact constituted an inseparable part of the separateness or closeness manifested in the dyadic observations. The need for contact was more apparent in the daughters but ranged from closeness to rejection. The initial contact was experiential and playful for the dyads but was subsequently experienced by most of the mothers as unpleasant and threatening, and as a type of “intrusion” into their virtual body. The sense of contact and closeness in the virtual space was different and surprising for both (Dyad 16, Figure 1): “It was a scary moment, I wonder how she (the daughter) experienced it, to suddenly feel this physicalness differently.” The mother described discomfort, since the VR simulates physical contact but differently from the real world. By contrast, the daughter said: “I’m taller than you” which may have reflected the altered spatial perspectives and perceptual experiences in the immersive virtual environment where virtual positioning and viewing angles can distort height and the spatial dominance that she did not experience in actual reality (Zhang et al., 2022).

In another dyadic interaction (Dyad 13), the daughter’s avatar hugged the mother’s avatar throughout the VR experience, and toward the end it began to kiss her mother, accompanied by a pronounced childish voice, a behavior that caused the mother discomfort, although at first she complimented her on the painting: “…Wow, the waves are beautiful…,” but then continued in a high voice: “A. … move away from me.” A. continued to touch her mother although her mother expressed discontent. A then said: “I’m kissing you, so do not move…” which further aggravated the mother’s sense of discomfort. In the interview, the daughter related to the hugs: “It was fun hugging her from within the avatar, it felt funny.” When the avatar body of one daughter entered the avatar body of her mother, the daughter experienced this as entering her mother’s womb. One of the daughters declared that she was an embryo. In the interview the daughter said (Dyad 13): “It was weird entering mom’s body; it’s like going back to [being] a baby. Because once, when I was young in a bathrobe, I would go into her pocket.” The mother added: “That’s right, I would cover her with the bathrobe, and after the bath we would do body to body.” The daughter: “And also when I was born, when I was in your stomach… and when I grew, I would put my feet like that in your pocket… I want to do it again.

These attitudes toward the avatar’s body as a personal body, although it was no different than the other avatars in space, reflected the mother-daughter relations. Its location and movements in the VR space, alongside the need for closeness, created different modes of physical communication in this space, including dancing (Dancing avatars)^1^. In most of the dyads, the daughter began the joint dance, and she usually continued to dance and move alone.

The teleport tool was used primarily by the daughters, to escape “far away” from the shared space with the mother. In many dyads, this created a situation where the mother searched for her daughter and called for her to come back, leading to a type of recurring hide-and-seek or catch game.

**Figure 2:**
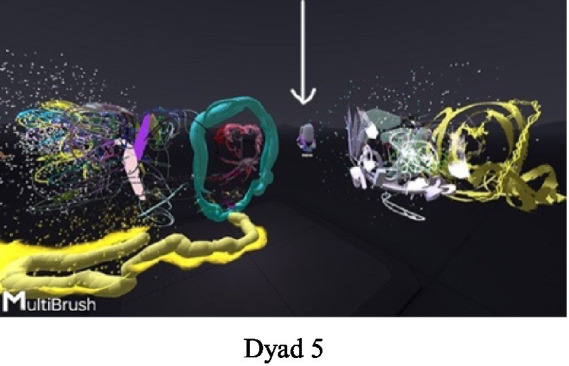
Don’t go, stay.

In the VR experience, one of the mothers asked her daughter to stay next to her (Dyad 5, Figure 2): “Do not go, stay, I will not move.” Despite her request, the daughter moved away, explaining: “It’s hard to paint things when they are so… close to you (to the mother) in the same area.” Another daughter asked her mother to go to another area so that she could be alone (Dyad 2): “Let us go to another room, to another space, and you move so that I can write….”.

One of the mothers stated that she felt almost entirely alone in the virtual space: “In 3D it was terribly hard to find each other in the space, so I felt that we were not painting together”.

#### Interpersonal-implicit (unconscious) communication in the virtual space

Non-verbal interpersonal communication between mothers and daughters in the virtual space also provided insights into their dyadic experiences. This communication revealed relations of presence and separation, primarily in the daughters. The joint experience of closeness and connection through technical questions, painting issues, and the mothers’ need for legitimization was gradually transformed into a need for distancing and separation. The dyadic process led to authentic rebellious self-expression by the daughters, using visual tools in the MultiBrush application such as creating cracks, holes, and transparent materials in the artwork, erasing, distancing themselves from joint space, forming enclosures using painting-related elements, and painting on the mother’s body. This was manifested at times using different painting positions, such as sitting and lying down. These provided additional cues to their dyadic relationship.

#### Cracks, holes, and transparent materials - expressing closeness versus space in the dyad

**Figure 3:**
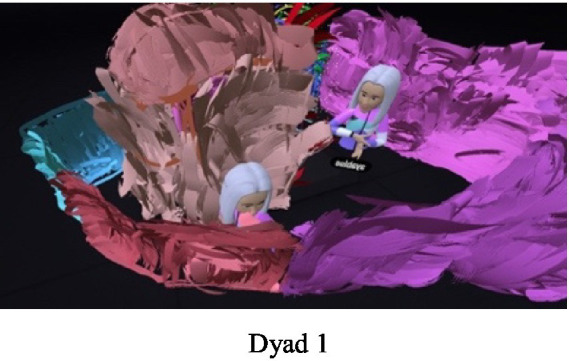
Building a fence together.

Part of the non-verbal communication corresponded to the felt need to fill the “holes” in the paintings using paint. One of the daughters said to her mother (Dyad 1, Figure 3): “Let us make a fence around everything we built… You (the mother) begin from the other side, until we meet,” and they began creating a “wall” around the joint painting. The daughter was anxious about the “holes” in the fence and noted that it was possible to break in through the holes: “We’ll have to check the whole fence to make sure that there are no holes,” and she went over to her mother’s side to verify this.

The issue of separateness was also apparent in paintings of transparent screens using transparent materials in VR that make it possible to paint in layers, see through objects, and create the illusion of space. The transparency of the 3D and movement materials alleviated the separateness by maintaining contact with the mother and allowing for the possibility of rapidly resuming a state of closeness (Scribbling enclosure)^2^.

#### Erasing to create separateness versus the desire for closeness - shifting power dynamics in the dyad

The erasure tool in the VR space makes it possible to erase parts of a painting. The daughters used the tool to create power positions and manifest aggressive behavior toward their mother while also making more room for themselves. At the beginning of the virtual experience, the erasure tool was used to the same extent by the mothers and daughters and served mainly to erase attempts that were not to the participants’ liking. At a later stage in the virtual experience, the daughters erased more, particularly when their mothers admired their paintings. In Dyad 14, for example, the mother, after complimenting her daughter on her artwork, was observed standing uncomprehending and asking: “You erased your painting… did you intend to erase it or…?” The daughter erased every time her mother liked her work. Another daughter erased the joint boundary between herself and her mother and asked (Dyad 1): “This is the boundary here, can we maybe erase the boundary?” and erased it without waiting for an answer. In other cases, the daughter erased entire segments of the mother’s painting throughout the virtual experience (Dyad 3). When asked in the interview what she had liked the most about painting in the joint space, she answered: “Painting on Mom!! And erasing things from her [painting] without her knowing. It’s fun.” She turned disparagingly to her mother and asked: “What would you like to tell me about the experience?”

A daughter in another dyad (Dyad 12) used the eraser many times. When her mother asked why she had erased, she answered: “I wanted to paint something that resembled yours, but I felt that the space was already taken.”

#### Scribbling and withdrawing into oneself

**Figure 4:**
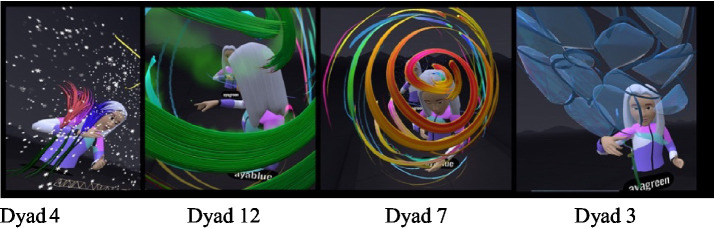
Transparent layers: creating distance while maintaining connection.

During middle childhood, one of the conflicts in mother-daughter relations has to do with the desire to separate and grow. This need was manifested in the VR space through scribbling and withdrawing into oneself (Figure 4).

##### Scribbling on mom

**Figure 5:**
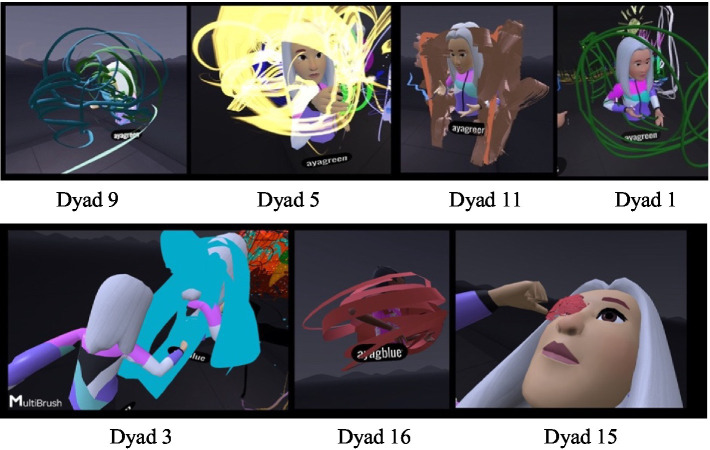
Erasing and marking mother: acts of resistance.

**Figure 6:**
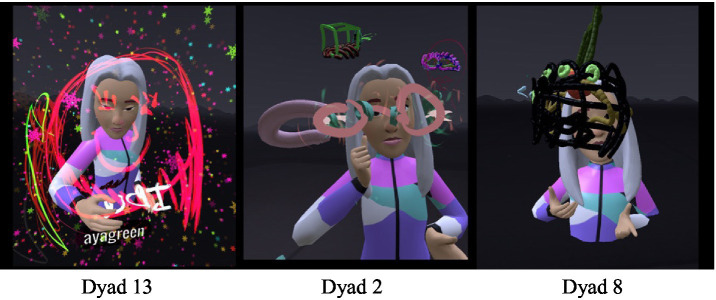
Painting mother’s face: between play and control.

In eight of the dyads, the daughters displayed a conspicuous need to scribble on their mother. For some, painting on the mothers was done aggressively and quickly, mainly in one color (Figure 5). In one case, the daughter explained she was painting a “scarf” on her mother as a joint painting (Dyad 15). In the interview she said: “It was fun. I admit that I took over, but that’s because she… it takes her a whole year….” The mother supported her: “…She takes command of everything. But it’s fun to see her.” Some of the daughters painted mainly on their mothers’ face, claiming it was part of a joint game (Figure 6). One of the mothers also related to her daughter’s painting on her as joint play and noted (Dyad 8): “…She (the daughter) spread paint on me and I evaded her and erased it,” and then added: “The interaction between us was that she (the daughter) picked on me, I escaped a little, and then erased it for her again”.

##### Withdrawing into oneself by enclosing the self or confining the mother

**Figure 7:**
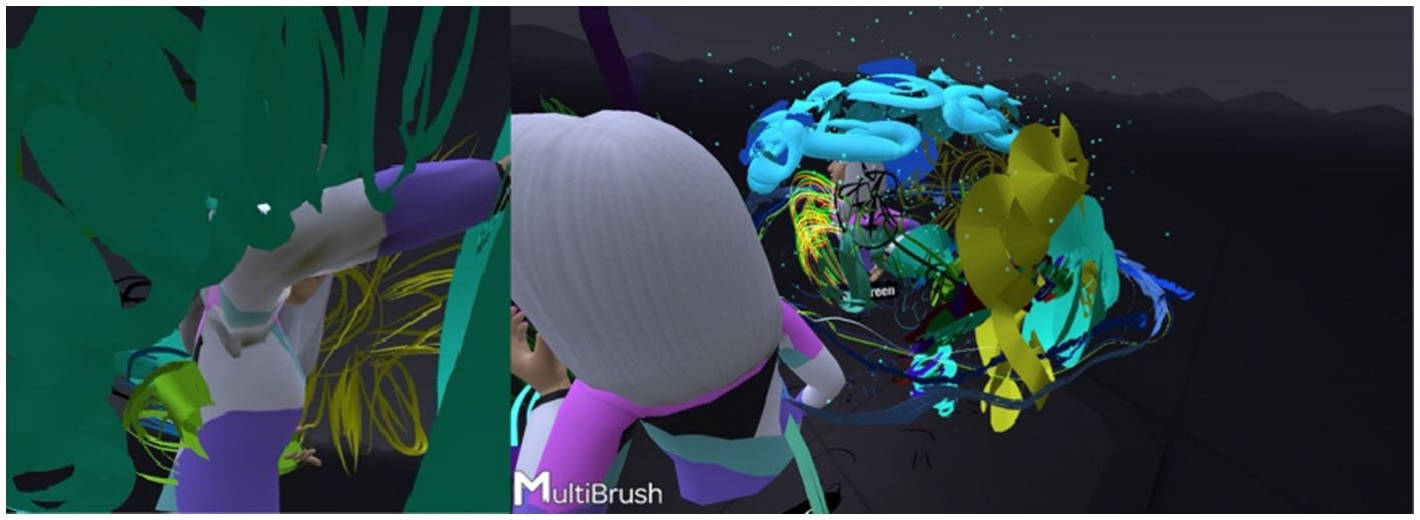
I’m in my space.

The daughters also expressed a need for separateness by withdrawing into themselves, and the virtual “confinement” of the mothers. For example, in one of the dyads the daughter “confined” the mother within a shape (Dyad 16): “I am doing this around you, you are confined.” In another dyad the daughter created a self-enclosure in a private “scribbled bubble” and declared (Dyad 13, Figure 7): “…I’m in my space and I cannot leave it.” This pattern recurred in other dyads where there were arguments throughout the art-based virtual experience. For example, in (Dyad 15) the daughter began to paint a scribble that enclosed the mother and said: “You’re blocking the paper for me… doing this on my face.” Daughter: “Wow, what a mess, you are closed in, I cannot see anything, you have to be erased.” This pattern of behavior made it possible to see the mother but prevented the mother from entering or seeing the daughters. The scribbling process, which involves rapid movement energy may have also provided emotion regulation.

#### Body postures when painting in VR - closeness versus distance and emerging mother-daughter conflict

In VR, standing and moving differ from actions in the real world. In the virtual space, painting is done mostly standing up while moving in space. Variations in standing and moving in the virtual space were considered indications of the ways in which body postures affected the mother-daughter interactions. The painting postures were thus analyzed as a type of relational indicator for the connection in the dyadic relationship. Body postures when standing were primarily face-to-face and back-to-back.

**Figure 8:**
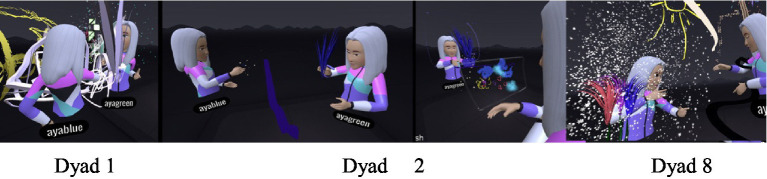
Face to face position.

Dyads who painted face-to-face (Figure 8) communicated more, mainly when planning the joint space, while they consulted and supported each other. In the interview, one mother (Dyad 1) said: “I felt more comfortable painting together with her in the joint space than alone. When I was alone, I felt like I did not really know what to paint and it was a little harder for me. With her [her daughter] it was easier, and I felt better.” The face-to-face position did not necessarily increase the amount of verbal communication but allowed the dyads to have a sense of eye contact and security. In addition, this position was accompanied by more two-way communication (Dyad 15). “How do you paint a bird?” “Mom, guess what I’m painting.” Most of the dyads who painted face-to-face appeared to have had a more secure experience in each other’s presence and showed much less of a need for separateness.

**Figure 9:**
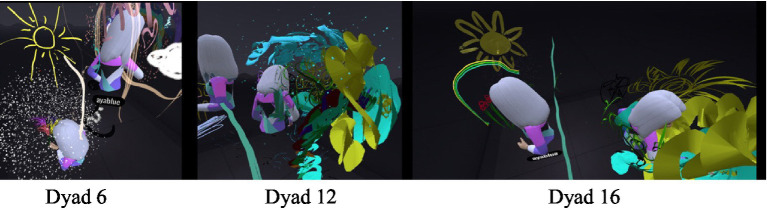
Back to back position.

In the dyads who painted back-to-back (Figure 9) or at a considerable distance from each other, the need for disconnecting and distancing was more apparent from the outset. Turning one’s back on the other may have been a manifestation of difficulties in communication. For example, one of the daughters declared (Dyad 5): “…I’ll go back to make it a little inside…”; she found a distant corner and asked the researcher to increase the distance even more: “Is there something not so close? Can I go anywhere I want?” Another example occurred at the start of the VR, when a mother and daughter stood back-to-back. The researcher asked them to turn around for a moment and drew the boundary line. The daughter then turned her back once again; later, when asked by her mother (Dyad 5): “Tuti, can I ask what you are painting?” the daughter answered: “No!!! You’ll see at the end!” Later on, her mother made further attempts to come closer but was answered aggressively: “Do not look!!” In the interview, the mother was asked whether the virtual experience was a good reflection of their relationship, and she answered (Dyad 5): “I think it’s pretty representative… I did not really look at what she did”.

### Second theme: 3D painting in VR and components of the mother-daughter relationship

The VR tool can generate infinite forms of expression and communication through representations that move in space, and an unlimited range of colors and textures. In this study, the MultiBrush application afforded considerable self-expression using diverse techniques. Note, however, that the content of the paintings was examined mainly from a phenomenological perspective in terms of the recurring elements, such as representations of a house, the images in the paintings, and the use of text.

#### The three-dimensional house

**Figure 10:**
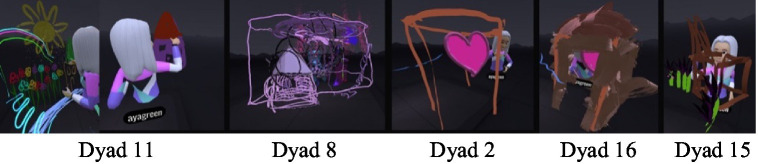
Entering the 3D house.

Many daughters painted a house in their personal space. These houses were initially done in 2D and then turned into 3D so it could be entered (Figure 10). This allowed for physical distancing, seclusion, and separateness. Most of the houses were made during the individual painting phase and were then erased. In one dyad (Dyad 11), the daughter painted a 3D house, entered it, added a roof, and wrote “my house.” She declared enthusiastically: “I made a house; I made a house.” The mother did not respond, because she was caught up in her own art. This prompted the daughter to paint another house “…something else, I’m changing the house.” In the interview the mother commented that: “She painted a house… I said okay, I know that she’s worried about us moving from the area… We’ve been living here for 14 years in a small house, but we have no intention of leaving it”.

**Figure 11:**
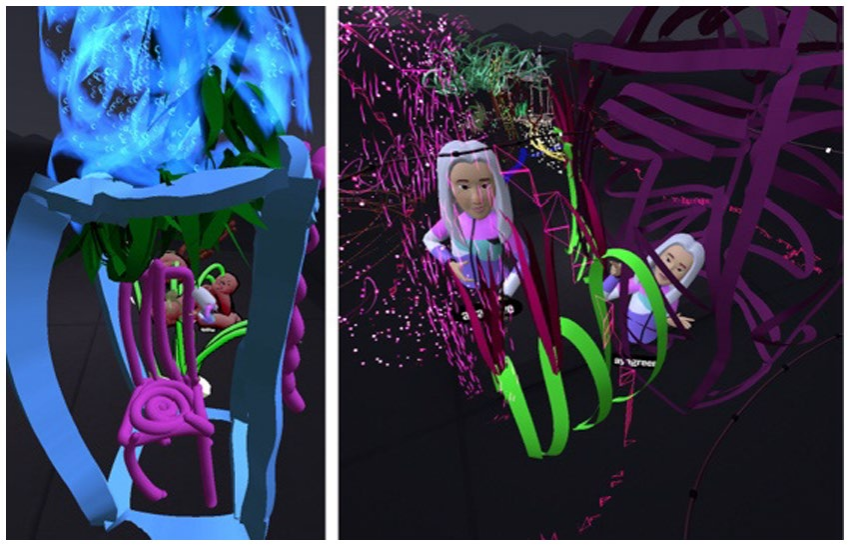
Merging spaces: from individual home to a shared virtual house.

In one of the dyads, the overweight daughter of a single mother painted a very large house in response to the mother’s small house and said, during the virtual experience (Dyad 8): “If only I had a house I could enter, that I could make….” In VR, the mother’s house was small and nicely designed, with two paths leading to it. She said that the back path was the daughter’s entrance. The daughter continued to paint her house throughout the virtual experience and to mutter: “I have a house that I can enter.” Subsequently, she decorated the house, while from time to time asking her mother: “Is my house pretty?” Eventually, she decided that the house was ugly. When it was her turn to enter her mother’s house she said: “But your house is small!” The mother responded by agreeing and said that they would remain in the yard. When the time came to name the painting, the mother suggested a title that contained all the letters from the names of the family members. The daughter fidgeted uncomfortably and said: “I do not know… me inside your house, it’s so small….” The mother interrupted: “And you are so big!!” The daughter ended the conversation by declaring: “So are you.” The daughter’s house subsequently became the focus of the dyadic interaction. Their houses were decorated with gates, windows, roofs, a fence, furniture and sparkling walls were added (Figure 11). then flowers were added, and became the “shared house.” After painting two separate houses, the daughter suggested to her mother that they work together (Dyad 1): “Let us make furniture and design the house.” The mother agreed: “Well, where do you want it? Let us get a little closer?.” Despite painting the furniture and decorating the house, the daughter maintained her distance from the mother and emphasized: “Let us work farther away so we can build more things”.

#### Recurring images and integrating text

Most of the dyads reproduced several images that were for the most part initially painted two-dimensionally, but where entering them in the virtual space made them three-dimensional. Elements such as flowers, suns, and hearts were enhanced by special VR effects, such as twinkling stars and shining rainbows, the three-dimensionality made it possible to see the images tangibly and respond to them emotionally and interactively.

Most mothers noted their lack of artistic skill compared to their daughters. More than half the dyads repeatedly drew the same basic elements, such as suns, clouds, rainbows, and flowers. One mother declared from the beginning that she drew like a preschooler (Dyad 10): “I drew a child’s drawing.” The daughter: “What did you draw?” The mother: “Like children draw in nursery school.” The daughter: “What do they draw in nursery school?” The mother: “Suns, flowers, and clouds.” These components appeared as the main themes or additions in the joint painting stage.

**Figure 12:**
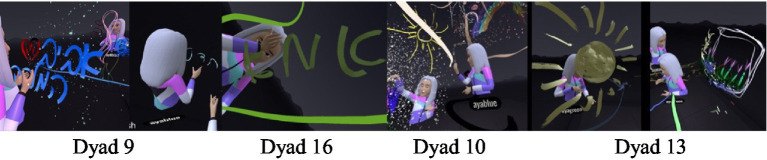
3D text as belonging and identity.

Text was mainly integrated into the artwork (Figure 12) by the daughters. The 3D text created a sense of belonging and pride in the artwork. The text was integrated primarily at the beginning of the virtual experience or at the end. For most of the dyads, the writing was used to be more specific and to give a clearer meaning to occurrences in the new unfamiliar space. Writing 3D text, whether a title, message, or name, may have allowed the dyads to undergo a more interactive experience and add content.

### Third theme: the immersive therapist in the dual therapeutic space

**Figure 13:**
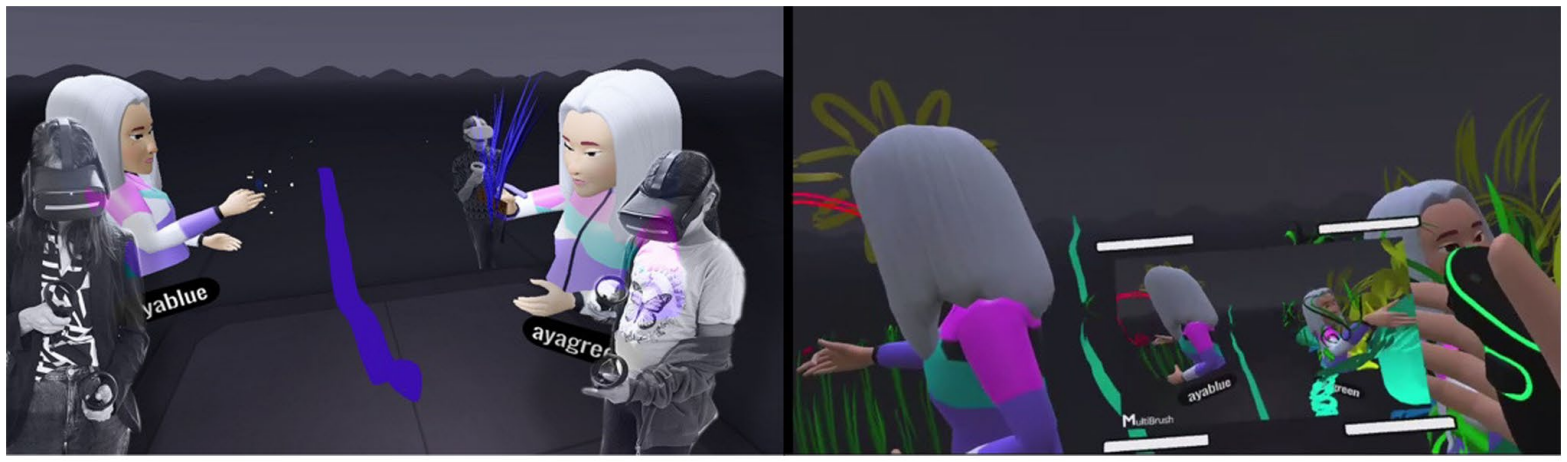
The immersive therapist.

The VR environment allows for the generation of an innovative therapeutic space where the therapist is inside the space with the dyad. The therapist can move and come close to the dyad, observe different angles and sizes, and experience the immersive experience together with the dyad. In the earlier versions of VR, the therapist observed the dyad through a two-dimensional screen, with no direct eye contact. In more recent applications, therapists can be in the physical world of the therapy room and in the virtual world. In dyadic therapy, the ability to enter a shared virtual therapeutic space in this case transformed the therapist from an observing participant into a 360-degree experiencing therapist (Figure 13).

Despite the presence of the immersive researcher in virtual space as the third avatar, most of the participants did not seem to notice her existence. The directions given by the researcher for transitioning between the stages of the procedure were experienced at times by the participants as interference or “awakening from a dream”; i.e., from their total experience. When asked in the interviews whether they felt the presence of the researcher or whether her presence had bothered them, one mother (Dyad 12) answered: …yours? No, not at all, we could only see within the frame and not look out, be inside… focus inwards….”.

Some of the participants interpreted the directions provided by the therapist and the reminders to transition from stage to stage as a warning that the procedure was about to end or as a concern that they might have to erase what they had already painted. For example, one of the dyads said that despite the researcher’s recurring directions, they disregarded them, and the mother only noticed that the instruction was aimed at them when the researcher raised her voice (Dyad 6): “One moment… Aya (the researcher) is talking to us.” The deep immersion in the virtual experience seems to have allowed the participants to focus on their relationship without being influenced by the researcher’s presence, for instance in Dyad 2:

…We could be one next to the other, each occupying herself with something… and she did not have to constantly say to me: “Mom, look.” Each one of us was immersed in her own [painting] and we were one next to the other, and we could engage in the same activity over time and enjoy ourselves… Like, for a moment we were sort of equal. Not in the role of mother and daughter… She occasionally said: “Mom, look,” and I turned around, but she was so excited that she nearly never said it, so I could be me. You? We did not see you (laughing).

When the immersive researcher was with the dyad in the 3D virtual space and observing their interactions, she felt the increased identification and transference. These feelings surfaced despite the absence of eye contact, since the participants were wearing VR headsets that prevented them from seeing one another. In the virtual dimension, the participants were represented as identical avatars, which bore no resemblance to their actual appearances, thus further precluding traditional visual interaction.

The researcher noted in her journal that:

Although the daughter asked her mother several times to confirm that the house she had painted was pretty and that the house that she [the mother] was painting was slightly too small for her, the mother did not respond and continued to paint a house that was smaller than the daughter’s dimensions. I felt a great deal of compassion for the daughter and anger at the mother who was immersed ‘in her pretty little model house’ (Dyad 8). I reminded myself that this is an exploratory procedure… and of my role as the researcher.

Thus, the researcher’s presence in the shared space with the dyad, as a facilitator and observer, appeared to have evoked intense projective material.

## Discussion

This study examined mother-daughter relations in middle childhood through a novel VR adaptation of the Joint Painting Procedure. To the best of our knowledge this is the first study of the integration of joint VR space into dyadic assessment where the therapist functions as an avatar within the same space. These exploratory findings with a nonclinical sample can best be interpreted within the developmental context of middle childhood, which is characterized by cognitive expansion, growing independence, and emotional–social consolidation (Collins and Madsen, 2019; Gilmore and Meersand, 2014). The VR-JPP interactions revealed three key relational processes: proximity versus distance, autonomy versus dependence, and conflict versus cooperation. Importantly, the observed conflicts represent normative developmental processes essential for healthy individuation rather than dysfunction (Brumariu and Kerns, 2022). The virtual environment provided a unique space for both revealing and processing these complex dynamics safely.

### The VR-JPP tool in virtual space – the immersive body, with no eye or physical contact

In the VR space, the participants were present in the physical and virtual space at the same time. In the virtual space the interaction took place with no eye or physical (tactile) contact with each other. The absence of physical contact in the mother-daughter dyads allowed them to focus on their emotional and creative expression with no physical distractions. However, the dyads tried to make contact, such as hugs through their avatars (Hadjipanayi et al., 2023; Zhang et al., 2023). The virtual space allowed more freedom for creative and emotional expression through images and movement (Bakk, 2023).

Wearing the VR headset and manipulating the avatars enabled the dyads to interact, with no need for direct gaze. The headsets set the participants apart. The absence of physical eye contact reduced the potential sense of exposure, embarrassment, and judgmentalism that may have eliminated some of the psychological barriers and enabled more authentic “here and now” emotional expressions (Blascovich and Bailenson, 2011; Nowak and Biocca, 2003; Bailenson et al., 2008). This seems to have been facilitated by communication through the avatars that encouraged a sense of presence, and greater self-identification (Freeman et al., 2020). The participants treated the avatars as their actual bodies, as manifested in the movements and interactions they made that resembled reality, including movement in the space – at times even dancing. This suggests that virtual space may allow for freer physical expression. The transition between physical space to virtual space in the art-based intervention (VR-JPP) brought the dynamics of the mother-daughter relations typical of middle childhood to the fore, as the mothers appeared to note in the interviews.

Middle childhood is characterized by complex interactions with the environment and a desire for social independence, alongside varied emotional challenges (Beyers and Goossens, 2008; Wong and Nadeem, 2013). The findings here suggested that virtual space allowed for powerful, rapid, and authentic expression of the mother-daughter relationship dynamics. The auditory segments emphasized the importance of verbal communication for connecting and coordinating between the mothers and daughters. At the beginning of the intervention, slightly regressive verbal communication was observed. The mothers and daughters used their voices to mediate the new space through short dialogs focused on conveying technical information and to orient themselves, while expressing enthusiastic and liberating comments. Later, the discourse developed to include terms expressing closeness and affectionate phrases by the mothers, while the daughters used a small set of short words, mainly “Mom,” The mothers’ verbal communication focused on verifying that their daughters were indeed managing to carry out the task; specifically, painting the boundary line required in the first stage of the experience. This boundary served as a physical representation in the virtual environment and as a metaphorical concept.

Subsequently, there was a drop in verbal communication when both participants turned their focus inward to immerse themselves in their painting. This was accompanied by an increase in mumbling, singing, and self-talk. The absence of direct eye contact in the virtual space may have reduced feelings of exposure and judgment, thus making it easier for the participants to concentrate on themselves. Thus, there was a clear disparity between the emotional and experiential discourse within the virtual space that allowed for intuitive and less structured communication, and the cognitive, more deliberate discourse typically observed outside the virtual experience. VR may thus encourage more intuitive and authentic emotional expression that is less controlled, which may result from the sense of security provided by the virtual environment, and in particular the absence of actual physical interaction (Shen, 2023; Stallmann et al., 2022). Studies have found that virtual space can provide a secure space for dealing with emotional conflicts in family dyads and makes it possible to express feelings more experientially than in the traditional physical space (Stallmann et al., 2022).

The implicit (non-verbal) communication that took place in the virtual space through artmaking and movement might be indicative of the daughters’ desire for a personal space and their need for independence. In most of the dyads, the virtual experience evolved from initial closeness and connection to distancing and separateness. The closeness began with virtual hugs; the daughters sought virtual physical closeness “like being a baby again,” while the mothers experienced it at times as intrusive and as crossing boundaries. However, at times, this gradually transitioned to painting back-to-back. Some of the daughters created enclosed shapes around themselves or around their mothers with scribbling or bubbles, thus generating a private disconnected space. Others erased the boundaries and used the teleport tool to distance themselves from the shared space. Painting fences around the artwork, erasing the mother’s paintings, and the hide-and-seek games where the mothers had to look for their daughters may have reflected a desire for separateness and control of the personal space (Raz, 2022). The complexity of the dyadic dynamics and the daughters’ need to develop independence were also conspicuous at the end of the virtual experience, when most of the daughters continued to paint on their own, whereas most of the mothers chose to stop. This may be illustrative of middle childhood as a stage of transition from dependency to independence, where the daughters expressed their aspiration for social independence (Wong and Nadeem, 2013).

The VR tool constituted a play environment that could be viewed as serving as a type of “transitional space” (Winnicott, 1971) forming a bridge between the participants’ inner worlds and the external world. The intermediate space, according to Winnicott, constitutes an area of subjective experience, where personal meaning is constructed in interaction with external reality. This allows for simultaneous separation and nearness to the world and the other through play and artmaking. At times, the virtual space also appeared to serve as a potential arena reflecting the power relations within the dyad (Liu and Chang, 2018), where the conflicts between the mothers and daughters were manifested in “playful artmaking.” The virtual space seemed to have allowed them to express their feelings more freely and securely through play and creativity.

This environment enabled articulations and behaviors that are impossible in the real world and thus provided new opportunities for exploring relationship dynamics. The use of visual techniques and tools such as erasing, creating peek holes, transparent materials, and producing scribbles and both transparent and opaque withdrawals provided a channel for expressing complex emotional needs. For example, one of the daughters was concerned that “holes” would appear in the shared fence. This may be suggestive of conflicting needs for protection and security, and fear of intrusion vs. a loss of independence. The daughters tangibly reflected the tension between the need for closeness and the urge for autonomy (Beyers and Goossens, 2008) through virtual transparent materials such as smoke, clouds, stars, and transparent virtual shapes. This allowed them to produce a personal space but still maintain “avatar eye contact” in the virtual space, which seemed to serve as a bridge between the need for separateness and the desire to maintain an emotional relationship and a sense of closeness and security between mother and daughter.

Scribbling and withdrawing into oneself could have also been a way to express the need for separateness and a mechanism for emotion regulation. The daughters created “scribbled bubbles” that allowed them to observe their mothers from a protected space while maintaining separateness. Winnicott suggested that scribbling may express unconscious urges and a transitional space where children combine their inner world with external reality (Winnicott, 2005). For the daughters, scribbling may have been a type of play that provided active escapism so that they could enter a regressive state within the play experience. The scribbling seems to have led spontaneously to primary games such as hide-and-seek that intensified rapidly at the pace of the immersive world. While playing, the daughters could express the desire to hide and to reveal themselves while dealing with the conflict between the wish to be exposed and concerns over it. Scribbling, like the “peek-a-boo” game, could have provided an opportunity to experience a sense of control over the external world, thus allowing the daughters to examine the boundaries between separateness and closeness.

The mother-daughter relations tended to include power struggles, where the mother attempted to maintain closeness and control while the daughter sought to define separate limits for herself (Friedman A., 2013) The “confining” of one of the mothers within a shape may represent a radical example of the desire to control the mother or restrict her. This may have reflected the daughter’s unconscious fear that her mother could take over her personal space.

Overall, the findings in this non-clinical sample may suggest that virtual space could enhance and accelerate the clinical treatment of the dynamics of closeness and distance in dyadic parent–child relationships. The artwork and the interviews both demonstrate the potential of VR-based therapy, which enables safe and creative transitional space for forming relationships and the visual expression of internal processes (Hacmun et al., 2021). The findings showed that the daughters tried to navigate between the need for closeness and support and the desire for separateness from their mothers. The erasure tool seems to have created the technological possibility to “erase the mother,” a conflict that is at the heart of the mother-daughter relations in middle childhood and expressed conflicts. The daughters used it not only to erase parts of their paintings but also as a way of getting attention and expressing frustration or resistance. For example, one of the daughters declared herself the “queen of erasures” and consistently erased parts of her mother’s painting while the mother repeatedly tried to paint. This behavior reflects a struggle for autonomy and separateness and is consistent with Gavron’s (2019) argument that joint artmaking encourages psychological processes and the acquisition of insights as also noted by Hacmun et al. (2021).

Previous studies using the Joint Painting Procedure have demonstrated that implicit dyadic expressions such as mutual recognition and low role confusion predict children’s social and academic adjustment, thus underscoring the value of capturing non-verbal relational processes (Gavron and Mayseless, 2015). Research has also shown that joint art-making generates relational dynamics such as mutual regulation, mentalization, and transformation, highlighting therapeutic-affective dimensions (Gavron and Mayseless, 2018). The VR adaptation here extends these findings by demonstrating how virtual environments can amplify such implicit processes through enhanced spatial manipulation and immersive presence.

### Body positions when painting in VR: face-to-face, back-to-back, flexible movement and distances

The body positions utilized in the virtual space provided interesting insights into the dyads’ relationship dynamics. Two main positions were observed while painting: face-to-face and back-to-back, and most of the dyads painted standing up. The face-to-face position allowed continuous successive communication, particularly when planning the joint space. The feeling of “virtual eye contact” (despite the lack of physical contact) seems to have enabled cooperation that resulted in a positive experience, and a smoother and more creative flow in the phases of the JPP, as well as “quality time,” as noted by one of the mothers. In contrast, although some of the dyads who stood face-to-face experienced a sense of security and unthreatening presence, the encounter at times led to two-way exchanges with many challenging questions and answers.

The back-to-back position appears to have reflected a need for disconnecting and distancing, sometimes as of the beginning of the JPP when defining personal boundaries in the intervention. This attitude mostly indicated complications and limited communication in the dyad and was often accompanied by aggressive reactions and defensiveness on the part of the daughters (Kedem et al., 2021). These findings may point to the value of VR as a tool for exploring parent–child relationships. In contrast to the external world, virtual space allows more flexible mother-daughter distance and movement. The choice of different body positions may provide insights into the emotional and communication needs of the dyad by reflecting and perhaps influencing the quality of the mother-daughter interactions and communication. In addition, it provides new insights into their relationship dynamics, which often cannot be observed in the real world (Kedem and Regev, 2021).

### Three-dimensional painting in VR as manifested in mother-daughter relations: mutual emulation, the use of text to express identity and belonging, and the image of the house as a space for separateness and autonomy

The three-dimensional painting in VR gave the dyads an opportunity to evaluate implicit components of their mother-daughter relations. Most daughters made a house when painting in the personal space, first in 2D and then in 3D so that they could enter it. The 3D house may have expressed a desire for separateness and autonomy, a process compatible with development in middle childhood (Gilmore and Meersand, 2014; Winnicott, 1971).

The findings also revealed recurring elements such as flowers, rainbows, suns, and hearts, which appeared in nearly all the dyads’ paintings and were further accentuated by special effects such as twinkling and movement (unique features of the VR environment). At the start of the intervention, the daughters had little confidence in their painting and tended to emulate their mothers. This was also evident when the daughters’ experienced difficulties with their painting, that prompted them to copy elements from their mothers’ drawings. This might reflect the attachment dynamics, where children tend to emulate their attachment figures, in this case the mother, as part of building confidence and connections (Rodgers et al., 2020b).

The daughters made the most use of text, at the beginning and end of the virtual experience; this seems to have been used to instill a sense of belonging and pride. At the beginning, the daughters wrote their names in large letters, and at the end gave a title to the joint painting. This use of text appears to have reflected independence and control of the dyadic relationship, but in some dyads, it also led to power struggles, particularly related to the choice of a title for the joint painting (Becht et al., 2019).

### The immersive therapist

VR technology forms an innovative therapeutic space where the researcher is assimilated into the same virtual space as the dyad. The findings showed that the presence of the therapist, represented as an avatar in the VR environment in addition to the dyad, did not disturb the interaction between the participants, who reported a deep and authentic experience despite her presence. Previous studies have indicated that the lack of physical contact and direct eye contact in the virtual space reduces feelings such as embarrassment and social pressure and contributes to enabling and exposed freedom of emotional expression (Blascovich and Bailenson, 2011; Nowak and Biocca, 2003).

The immersive therapist, who was “invisible,” did not interfere with the projective material that surfaced in the dyadic relationship. It was clear from the researcher’s later comments that her presence in virtual space also allowed her to experience transference and countertransference differently than in traditional therapy. Specifically, despite being an observer, the researcher experienced transference in a direct and emotionally enhanced way, thus raising the possibility that her presence as an avatar, rather than as a physical figure, allowed her to remain neutral, since she was perceived as a virtual avatar rather than a physical figure. This allowed her to participate in space passively, without interfering in the transference occurring between mother and daughter. This immersive presence underscores the importance of neutrality in the transference that occurs in therapy where the therapist’s physical presence can affect the emotional dynamics (Ogden, 1994; Gabbard, 2001).

These observations align with emerging research on group-based VR therapy, where the therapist’s presence in virtual spaces has been found to maintain therapeutic efficacy while reducing social pressure. Studies on VR group therapy have shown that avatar-mediated anonymity can increase participants’ willingness to disclose and can improve group cohesion (Dilgul et al., 2020). Similarly, studies on multi-user VR experiences have reported greater effectiveness in improving mental health outcomes among adolescents in terms of higher engagement rates and the formation of meaningful social connections (Li and Shen, 2022). These findings suggest that the immersive therapist role, as examined in VR-JPP, may extend beyond dyadic interventions and could inform future development of group-based and community VR applications in art therapy. Recent work in HCI further points to the potential of multi-user VR for both assessment and therapy, by highlighting how shared virtual environments can enhance therapeutic presence and dyadic as well as group-level engagement (Haeyen et al., 2021a).

## Limitations and directions for further research

This study has several limitations. VR-JPP is an innovative tool, and further studies are needed to establish its validity and reliability. Another limitation is the homogeneity of the sample since this study focused solely on dyads of nonclinical mothers and daughters in middle childhood. This considerably limits the generalizability of the findings to other populations, such as dyads of mothers and sons, fathers and children, or differently structured families. The effects of technological innovation also need to be considered, since the participants’ lack of technological experience might have affected the quality of the experience. The participants’ reactions may have been affected by the innovative nature of VR so that their exchanges may not have necessarily reflect natural interactions. In addition, the researcher’s bias in terms of her active involvement as an “immersive therapist” might have affected the participants’ behavior and the interpretation of the findings.

Further studies should be conducted to establish the efficacy and applications of the VR-JPP as a psychotherapy and assessment tool for examining parent–child relationships. Giving participants the choice of using personal avatars should be examined and the role of the immersive therapist and the effects of the therapist’s presence in the shared space on therapeutic processes should be investigated.

The current study constitutes a first step toward developing a VR-JPP tool and testing the use of VR in dyadic therapy. More research is needed to confirm the efficacy of the tool, examine its effects on dyadic relationships, and develop standard protocols for its use. The findings also pave the way for diagnosis and therapy for the new generation whose quality of attention and interest are impacted by advanced technology and rapid reactive speed. Future studies could enrich interpretations by incorporating psychophysiological measures such as heart rate variability or galvanic skin response to better capture dyadic effects and engagement in virtual environments. In addition, while VR-JPP adapts the established JPP framework, further validation against traditional JPP outcomes is warranted. Previous research has demonstrated that implicit processes such as mutual recognition and reduced role confusion are strongly associated with children’s social and academic adjustment (Gavron and Mayseless, 2015), and that joint art-making can elicit transformational therapeutic experiences (Gavron and Mayseless, 2018). Future work should examine how the VR-mediated format enhances or alters these relational and therapeutic mechanisms. Finally, building on recent advances in VR mental health interventions, VR-JPP may be extended in several innovative directions: (a) multi-dyad VR environments enabling peer support and inter-family learning; (b) AI-enhanced personalization using biofeedback for real-time adaptation; and (c) systematic evaluation of freely available VR content to expand therapeutic accessibility.

## Data Availability

The original contributions presented in the study are included in the article/supplementary material, further inquiries can be directed to the corresponding author.

## Footnotes

^1^ https://docs.google.com/document/d/1mUBNanTQop19_mYDzQEQiPdkZfDbxM7OwUXHrFUQmmw/

^2^ https://docs.google.com/document/d/1IXZdUHRJyOeBG4okASzsXKQrp1Lr4OowqG4sPL7CM3Y

## References

[ref1] Alsem S. C. van Dijk A. Verhulp E. Dekkers T. de Castro B. O. (2023). Treating children’s aggressive behavior problems using cognitive behavior therapy with virtual reality: a multicenter randomized controlled trial. Child Dev. 94, e334–e361. doi: 10.1111/cdev.13966 37459452

[ref2] Bailenson J. N. Yee N. Blascovich J. Beall A. C. Lundblad N. Jin M. (2008). Theuse of immersive virtual reality in the learning sciences: digital transformations of teachers, students, and social context. J. Learn. Sci. 17, 102–141. doi: 10.1080/10508400701793141

[ref3] Bakk A. K. (2023). Representing mental disorders with virtual reality applications: designing for multimodality and complex participation. Front. Virtual Reality 2023:1766. doi: 10.3389/frvir.2022.881766

[ref4] Bauer M. W. Gaskell G. (2000). Qualitative researching with text, image and sound: A practical handbook. Los Angeles: Sage Publications.

[ref5] Becht A. I. Luyckx K. Nelemans S. A. Goossens L. Branje S. J. Vollebergh W. A.. (2019). Linking identity and depressive symptoms across adolescence: a multisample longitudinal study testing within-person effects. Dev. Psychol. 55:1733. doi: 10.1037/dev0000742 31070434

[ref6] Best P. Manktelow R. Taylor B. J. Taylor J. (2022). Evaluating the use of freely available virtual reality content for mental health practice: opportunities and challenges. Front. Virtual Reality 3:834567. doi: 10.3389/frvir.2022.834567

[ref42] Beyers W. Goossens L. (2008). Dynamics of perceived parenting and identity formation in late adolescence. J. Adolesc. 31, 165–184. doi: 10.1016/j.adolescence.2007.04.003 17629552

[ref7] Blascovich J. Bailenson J. N. (2011). Infinite reality: Avatars, eternal life, new worlds, and the dawn of the virtual revolution. New York: William Morrow.

[ref8] Bornstein M. H. Putnick D. L. Suwalsky J. T. (2022). “A longitudinal process analysis of mother-child emotional relationships in a rural Appalachian European American community” in Parenting: Selected writings of Marc H. Bornstein. ed. Bornstein M. H. (New York: Routledge), 232–253. 10.1007/s10464-011-9479-1 PMC3424281 22080397

[ref9] Brumariu L. E. Kerns K. A. (2022). “Parent–child attachment in early and middle childhood” in The Wiley-Blackwell Handbook of Childhood Social Development. eds. Smith P. K. Hart C. H. (Hoboken, NJ: Wiley), 425–442.

[ref10] Charmaz K. (2006). Constructing grounded theory. Newcastle upon Tyne: Sage.

[ref11] Collins W. A. Madsen S. D. (2019). “Parenting during middle childhood” in Handbook of parenting. ed. Collins W. A. (Abingdon: Routledge), 81–110.

[ref12] Day B. Ebrahimi E. Hartman L. S. Pagano C. C. Robb A. C. Babu S. V. (2019). Examining the effects of altered avatars on perception-action in virtual reality. J. Exp. Psychol. Appl. 25:124. doi: 10.1037/xap0000192 30346194

[ref13] Dilgul M. Martinez J. Laxhman N. Priebe S. Bird V. (2020). Cognitive behavioural therapy in virtual reality treatments across mental health conditions: a systematic review. Consort. Psychiatr. 1, 30–46. doi: 10.17650/2712-7672-2020-1-1-30-46, PMID: 38680386 PMC11047275

[ref14] Fisher Grafy H. (2015). *Socially rejected children: Theory and therapy*. Resling.

[ref15] Freeman G. Zamanifard S. Maloney D. Adkins A. (2020). *My body, my avatar: how people perceive their avatars in social virtual reality*. In: Extended abstracts of the 2020 CHI Conference on Human Factors in Computing Systems, pp. 1–6.

[ref16] Friedman M. (2013). Parent–child relations in adolescence. Abingdon: Routledge.

[ref17] Friedman A. (2013). *Mothers and daughters: An entangled relationship*. Hakibbutz Hameuchad.

[ref18] Gabbard G. O. (2001). A contemporary psychoanalytic model of countertransference. J. Clin. Psychol. 57, 983–991. doi: 10.1002/jclp.1065 11449380

[ref19] Gavron T. (2013). Meeting on common ground: assessing parent-child relationships through the joint painting procedure. Art Ther. 30, 12–19. doi: 10.1080/07421656.2013.757508

[ref20] Gavron T. (2017). The joint painting procedure: parents and children creating shared space. Art Therapy Online 8:428. doi: 10.25602/GOLD.atol.v8i1.428

[ref21] Gavron T. (2019). The joint painting procedure (JPP): The rating manual. Haifa: University of Haifa, Emili Sagol.

[ref22] Gavron T. Feniger-Schaal R. Peretz A. (2022). Relationship aspects of mothers and their adolescents with intellectual disability as expressed through the joint painting procedure. Children 9:922. doi: 10.3390/children9060922 35740859 PMC9221804

[ref23] Gavron T. Mayseless O. (2015). The joint painting procedure to assess implicit aspects of the mother–child relationship in middle childhood. Art Ther. 32, 83–88. doi: 10.1080/07421656.2015.1028007

[ref24] Gavron T. Mayseless O. (2018). Creating art together as a transformative process in parent-child relations: the therapeutic aspects of the joint painting procedure. Front. Psychol. 2018:2154. doi: 10.3389/fpsyg.2018.02154 PMC6243114 30483180

[ref25] Gilmore K. J. Meersand P. (2014). The little book of child and adolescent development. Oxford, UK: Oxford University Press.

[ref26] Gilmore K. J. Meersand P. (2023). Emerging adulthood: A psychodynamic approach to the new developmental phase of the 21st century. Washington, DC: American Psychiatric Pub.

[ref27] Hacmun I. Regev D. Salomon R. (2018). The principles of art therapy in virtual reality. Front. Psychol. 9:2082. doi: 10.3389/fpsyg.2018.02082, PMID: 30429813 PMC6220080

[ref28] Hacmun I. Regev D. Salomon R. (2021). Artistic creation in virtual reality for art therapy: a qualitative study with expert art therapists. Art Psychother. 72:101745. doi: 10.1016/j.aip.2020.101745

[ref29] Hadjipanayi C. Banaku D. Michael-Grigoriou D. (2023). Art as therapy in virtual reality: a scoping review. Front. Virtual Reality 4:1065863. doi: 10.3389/frvir.2023.1065863

[ref30] Haeyen S. Jans N. Glas M. Kolijn J. (2021a). VR health experience: a virtual space for arts and psychomotor therapy. Front. Psychol. 12:704613. doi: 10.3389/fpsyg.2021.704613 34594268 PMC8476779

[ref31] Haeyen S. Jans N. Heijman J. (2021b). The use of VR tilt brush in art and psychomotor therapy: an innovative perspective. Arts Psychother. 76:101855. doi: 10.1016/j.aip.2021.101855

[ref32] Hart J. Piumsomboon T. Lee G. A. Smith R. T. Billinghurst M. (2021). *Manipulating avatars for enhanced communication in extended reality*. IEEE International Conference on Intelligent Reality (ICIR), 2021, pp. 9–16.

[ref33] Jans N. Wouters H. Kolijn J. Haeyen S. (2025). Developing a virtual reality application for online arts and psychomotor therapies using action research. Front. Virtual Reality 6:1462775. doi: 10.3389/frvir.2025.1462775

[ref34] Kaimal G. Carroll-Haskins K. Berberian M. Dougherty A. Carlton N. Ramakrishnan A. (2020). Virtual reality in art therapy: a pilot qualitative study of the novel medium and implications for practice. Art Ther. 37, 16–24. doi: 10.1080/07421656.2019.1659662

[ref35] Kapitan L. (2010). Introduction to art therapy research. London: Routledge.

[ref36] Kaplan H. (2019). *Dyadic therapy: Thoughts from the therapy room*. Pardes.

[ref37] Kedem D. Regev D. (2021). Parent-child dance and movement therapy (PCDMT): mothers’ subjective experiences. Body Movement Dance Psychother 16, 136–149. doi: 10.1080/17432979.2021.1883740

[ref38] Kedem D. Regev D. Guttmann J. (2021). Moving together: assessing the effectiveness of group mother-child dance and movement therapy. Arts Psychother. 74:101803. doi: 10.1016/j.aip.2021.101803

[ref39] Knowles J. G. Cole A. L. (2007). Handbook of the arts in qualitative research: Perspectives, methodologies, examples, and issues. Newcastle upon Tyne: Sage Publications.

[ref40] Leavy P. (2020). Method meets art: Arts-based research practice. New York: Guilford Publications.

[ref41] Lee S. Mun I. B. (2022). How does perceived parental rejection influence cyberbullying by children? A serial mediation model of children’s depression and smartphone addiction. Soc. Sci. J. 2022, 1–16. doi: 10.1080/03623319.2022.2070826

[ref43] Li B. Shen M. (2022). The psychological recovery of patients in the context of virtual reality application by a complementary medicine scheme based on visual art. Evid. Based Complement. Altern. Med. 2022, 1–5. doi: 10.1155/2022/7358597 PMC9526666 36193137

[ref44] Liu Y. Chang C. L. (2018). *The application of virtual reality technology in art therapy: a case study of tilt brush*. 1st IEEE International Conference on Knowledge Innovation and Invention, pp. 47–50. IEEE. Available online at: https://ieeexplore.ieee.org/stamp/stamp.jsp?tp=&arnumber=8569081.

[ref45] Liu Z. Q. Dance S. (1995). Picture interpretation: A symbolic approach (20). New Jersey: World Scientific.

[ref46] Lugrin J. L. Ertl M. Klupfel R. Stierstorfer S. Weisz B. Ruck M.. (2018). *Any “body” there? Avatar visibility effects in a virtual reality game*. 2018 UEEE Conference on Virtual Reality and 3D User Interfaces (VR), 17–24.

[ref47] Malchiodi C. A. (2012). Handbook of art therapy. 2nd Edn. New York: Guilford Press.

[ref48] McNiff S. (2008). Arts-based research. London: Jessica Kingsley Publishers.

[ref49] Nowak K. L. Biocca F. (2003). The effect of the agency and anthropomorphism on users’ sense of telepresence, copresence, and social presence in virtual environments. Presence 12, 481–494. doi: 10.1162/105474603322761289

[ref50] O’Connor C. Joffe H. (2020). Intercoder reliability in qualitative research: debates and practical guidelines. Int J Qual Methods 19:1609406919899220. doi: 10.1177/1609406919899220

[ref51] Ogden T. H. (1994). The analytic third: working with intersubjective clinical facts. Int. J. Psychoanal. 75, 3–19, PMID: 8005761

[ref52] Patton M. Q. (1990). Qualitative evaluation and research methods. 2nd Edn. Newcastle upon Tyne: Sage.

[ref53] Perez T. V. (2024). A digital social mirror for identity development during adolescence. Curr. Psychol. 43, 22170–22180. doi: 10.1007/s12144-024-05980-z

[ref54] Piryankova I. (2015). The influence of a self-avatar on space and body perception in immersive virtual reality (42). Berlin, Germany: Logos Verlag Berlin GmbH.

[ref55] Rahman M. Rahman S. M. M. (2021). *Synthesis of user-level 3D head avatar from smartphone video for virtual reality*. 2021 24th International Conference on Computer and Information Technology (ICCIT), pp. 1–6.

[ref56] Rasmussen D. M. (2012). Symbol and interpretation. Berlin, Germany: Springer Science and Business Media.

[ref57] Raz G. (2022). Rage against the empathy machine revisited: the ethics of the empathic affordances of virtual reality. Convergence 2022:406. doi: 10.1177/13548565221086406

[ref58] Rodgers R. F. Donovan E. Cousineau T. McGowan K. Luk S. Yates K.. (2020b). BodiMojo: efficacy of a mobile-based intervention in improving body image and self-compassion among adolescents. J. Youth Adolesc. 49, 1221–1235. doi: 10.1007/s10964-020-01210-8 29349593

[ref59] Rodgers R. F. Slater A. Gordon C. S. McLean S. A. Jarman H. K. Paxton S. J. (2020a). A biopsychosocial model of social media use and body image concerns, disordered eating, and muscle-building behaviors among adolescent girls and boys. J. Youth Adolesc. 49, 399–409. doi: 10.1007/s10964-019-01190-0 31907699

[ref60] Routledge Creswell W. J. (2014). *Research design: Qualitative, quantitative, and mixed methods approaches*. CPI Group.

[ref61] Rubin J. A. (2010). Introduction to art therapy: Sources and resources. Abingdon: Routledge.

[ref62] Sabar-Ben Yehoshua N. (2016). Research genres and traditions: Advanced tools, strategies, and philosophies. Tel Aviv: Mofet Institute.

[ref63] Shamri-Zeevi L. (2021). Making art therapy virtual: integrating virtual reality into art therapy with adolescents. Front. Psychol. 12:584943. doi: 10.3389/fpsyg.2021.584943 33613377 PMC7889518

[ref64] Shamri-Zeevi L. Kedem-Sarrabia D. (2023). Creating parenting: Art and movement based therapeutic interventions for parents. Haifa: University of Haifa, Emili Sagol Research Center.

[ref65] Shamri-Zeevi L. Regev D. (2021). Parental responses to artistic processes in art-based parental training. ArTherapy 38, 159–165. doi: 10.1080/07421656.2020.1816106

[ref66] Shamri-Zeevi L. Regev D. Guttmann J. (2015). The usage of art materials in the assessment and treatment of parental reflective functioning. Arts Psychother. 45, 11–18. doi: 10.1016/j.aip.2015.07.002

[ref67] Shen C. (2023). *Fostering empathy through intergenerational storytelling in embodied virtual reality [thesis]*. University of British Columbia. Available online at: https://open.library.ubc.ca/soa/cIRcle/collections/ubctheses/24/items/1.0435683.

[ref68] Spector-Mersel G. (2010). Time for a paradigm. Narrat. Inq. 20, 204–224. doi: 10.1075/ni.20.1.10spe

[ref69] Stallmann L. Dukes D. Tran M. De Gevigney V. D. Samson A. C. (2022). Socially supported by an embodied agent: the development of a virtual-reality paradigm to study social emotion regulation. Front. Virtual Reality 2022:241. doi: 10.3389/frvir.2022.826241

[ref70] Strauss A. Corbin J. (1990). Basics of qualitative research: Grounded theory procedures and techniques. Newcastle upon Tyne: Sage.

[ref71] Winnicott D. W. (1971). Playing and reality. Tavistock: Tavistock Publications.

[ref72] Winnicott D. W. (2005). Playing and reality. Abingdon: Routledge.

[ref73] Wong M. Nadeem E. (2013). “Responding to the challenges of preadolescence: roles for caregivers” in Child development in the social context. eds. Arnold S. H. Kaplan R. J. (Berlin: Springer), 47–59.

[ref74] XR Today. (2023). *The Meta Quest 3 Guardian: How to use the smart Guardian*. Available online at: https://www.xrtoday.com/mixed-reality/the-meta-quest-3-guardian-how-to-use-the-smart-guardian/.

[ref75] Zhang J. Dong Z. Bai X. He W. Lindeman R. Piumsomboon T. (2022). Augmented perception through spatial scale manipulation in virtual reality for enhanced empathy in design-related tasks. Front. Virtual Reality 3:672537. doi: 10.3389/frvir.2022.672537

[ref76] Zhang Z. Front J. M. Gimenez Mateu L. (2023). Facial expression recognition in virtual reality environments: challenges and opportunities. Front. Psychol. 14:1280136. doi: 10.3389/fpsyg.2023.1280136, PMID: 37885738 PMC10598841

